# Atypical Kawasaki Disease in a 4 Years Old Child with Mumps

**DOI:** 10.31729/jnma.3482

**Published:** 2018-08-31

**Authors:** Prakash Banjade, Kiran Subedi

**Affiliations:** 1 Department of Pediatrics, Kathmandu Medical College Teaching Hospital, Kathmandu, Nepal

**Keywords:** *bilateral cervical adenitis*, *fever and mumps*, *failed to respond IV antibiotics*, *incomplete kawasaki disease*

## Abstract

Kawasaki disease is an acute febrile condition seen in children. However, it is also well recognized that some patients do not fulfill the classic diagnostic criteria for the diagnosis of Kawasaki disease. The incomplete form of Kawasaki disease is termed as ‘Incomplete KD’ or ‘Atypical KD’. This is a case of 4 years old child with fever and mumps. He had bilateral cervical adenitis. Patient failed to respond to IV antibiotics fulfilled the criteria of incomplete Kawasaki disease. The child was managed with high dose aspirin until the child was afebrile for 48 hours. Kawasaki disease is a common vasculitis in children. Atypical cases might be missed if there is concomitant viral illness. Hence the identification and management of Kawasaki disease is paramount to decrease the mortality related to the cardiac disease.

## INTRODUCTION

Kawasaki disease is acute, self-limited systemic type of vasculitis that occurs predominantly in young children. After first case reported by Tomisaku Kawasaki in Japan,^1^ it is recognized as the leading cause of acquired heart disease in children in developed countries. However, pediatricians sometimes encounter febrile children who do not fulfill the diagnostic criteria but have several findings compatible with those of Kawasaki disease. In this situation, the diagnosis of incomplete Kawasaki disease is a clinical challenge. It cannot be avoided by delaying the diagnosis because of the risk of coronary complications pertaining even to the incomplete presentation of the disease.^2,3^

The reported prevalence of incomplete presentation was 15 to 36.2% among patients with Kawasaki disease.^4,5^ Children with incomplete presentation were more likely to be at the extremes of the age spectrum as compared to those with complete presentation. The prevalence of incomplete presentation was relatively higher in the younger-aged patients.^4,5^

## CASE REPORT

A 4 years old male child presented to hospital with swelling behind left ear and fever for 7 days. The child had been without any symptoms 7 days back. He started to have fever (maximum recorded temperature of 103^0^ F with no chills or rigors). He had swelling behind the left ear, painful (mild in intensity and relieved with analgesic medications) and difficulty in moving the neck forward and side to side. Swelling was pea sized and multiple in numbers including the neck bilaterally. Size of the swelling remained constant throughout. No history of history of right ear pain, ear discharge, redness of eyes, eye discharge, abnormal body movements, LOC, difficulty in swallowing, runny nose, cough, chest pain, fast breathing. He didn't have h/o pain while chewing, loose stool, nausea, vomiting, abdominal pain, increase frequency of urination, burning urination, blood in urine.

He had history of contact with friends having mumps. No history of joint swelling or rashes. He has cats at home sometimes plays with them but no history of scratch by them and no history of tick bite, and history of swelling below the ears as described by mother as mumps in the right side had subsided at the time of presentation. He had no history of dental caries no facial lesions, history of swelling of the right lower limbs ankle subsiding on its own.

He was treated with Azithromycin for 5 days with Cefixime 2 days already, when the child presented to hospital.

On Examination, child was alert and looked in no apparent distress but looked toxic.

**Figure 1.**
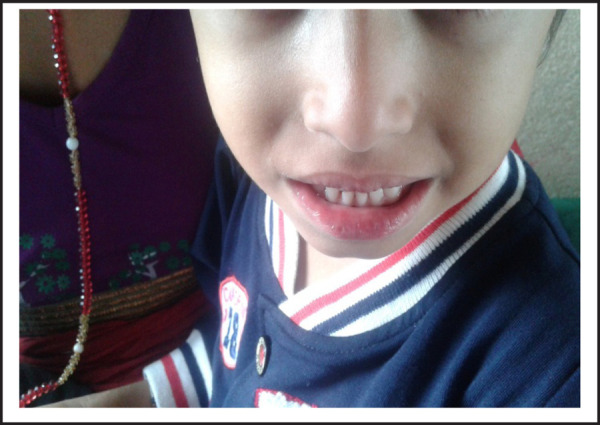
Mucosal changes and lip cracking.

**Figure 2.**
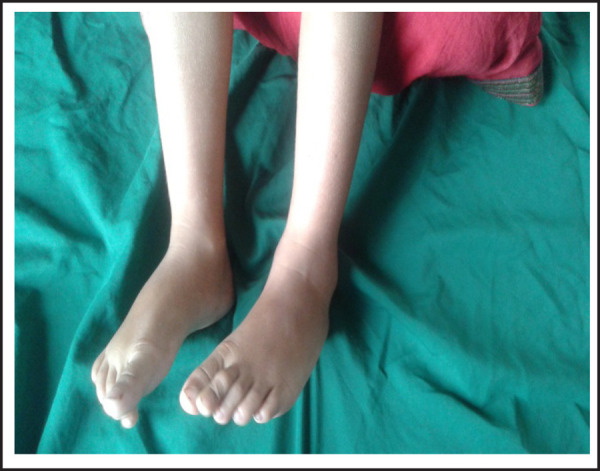
Edema of the feet, right ankle.

Temperature was 102^0^ F, axillary pulse rate was 110/ min with normal character, volume with no radioradial and radio-femoral delay. All peripheral pulses were palpable. Respiratory rate was 32 per minutes, SpO2 -94 % in right arm. BP was 100/60 mm Hg right arm supine. Pallor, icterus, cyanosis were absent. Edema and lymphadenopathy were present. Multiple, discrete, tender, sized less than 1 cm in pre-auricular, post auricular, submandibular and inguinal area were palpable. Axillary lymph nodes were not palpable.

On examination of head, there was no scalp lesion, hair was normal. Examination of eye revealed no redness and discharge. Ear examination revealed bilateral wax. Oral cavity examination showed no ulcers, normal pharynx, stenson's duct, no dental caries. Face examination reveal no rashes no lesions.

Investigations (on 7^th^ day of symptom) showed hemoglobin 10.8 gm/dl, total count 18,000/mm^3^ (neutrophils 65%, lymphocytes 35%), platelets-50,000/mm^3^, CRP present. Chest X-ray was normal. Urine microscopic examination were normal, viral panel EBV, mumps, adenovirus were sent in which mumps IgM was positive.

During the hospital stay till 3 days of treatment with IV antibiotics which was started in view of severe bacterial infection, the child remains febrile with temperature up to 103^0^F, so blood investigations were sent on 4^th^ day of hospital admissions and 11^th^ day of illness.

**Figure 3.**
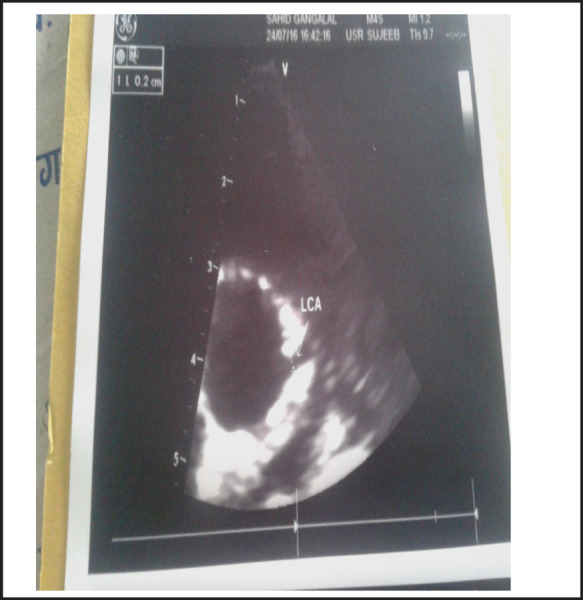
ECHO-on the left shows normal size Left coronary artery.

**Figure 4.**
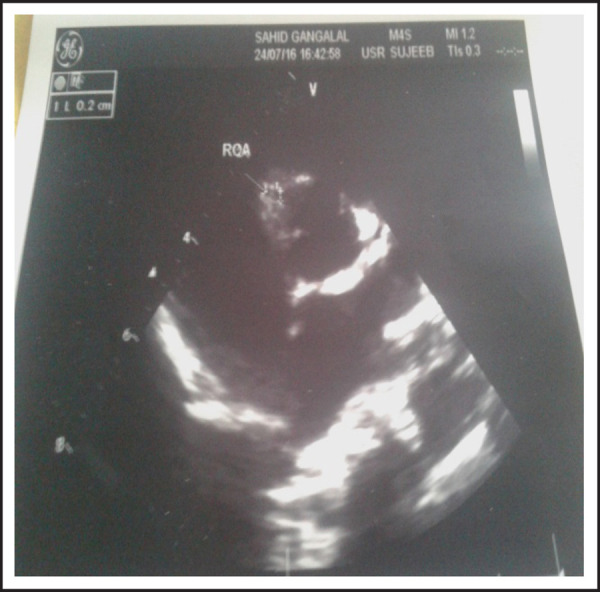
Normal size Right coronary artery in ECHO.

His baseline laboratory work-up were sent. Abnormal laboratory findings included a low hemoglobin (10.4 gm/dl), a low hematocrit, raised white cell count (21 x 109/L), with dominance of neutrophils (85%), and thrombocytosis (platelet count of 539 X 10^3^/mm^3^). The rest of the work-up including electrolyte and renal function workup was within the normal range. Hence a diagnosis of incomplete Kawasaki disease was made since few of the clinical features were met according to Japanese criteria^6^ along with the laboratory criteria's.

**Table 1:** Japanese criteria for diagnosis of Kawasaki disease.

Fever persisting ≥ 5 days
Bilateral conjunctival congestion
Changes of lips and oral cavity
Polymorphous exanthema
Changes of peripheral extremities
Acute non-purulent cervical lymphadenopathy

## DISCUSSION

Kawasaki disease is an acute febrile condition seen in children. Even though it was first reported in Japan about 30 years ago, the original diagnostic criteria defined by Dr. Tomisaku Kawasaki in 1967 are still authentic and widely used today. However, it is also well recognized that some patients do not fulfill the classic diagnostic criteria for the diagnosis of KD. The incomplete form of KD is termed as Incomplete KD' or ‘Atypical KD’. Because incomplete KD is not a mild form of KD, children remain at similar risk for cardiovascular sequelae as that of complete KD.^7^ Since the disease has a similar risk of coronary artery abnormalities (CAA) as complete KD,^8–10^ it is necessary to make an accurate diagnosis in order to prevent the development of coronary artery abnormalities CAA.

**Table 2:** Additional laboratory criteria and Echocardiography criteria according to Japanese criteria.^11^

(A)
Serum albumin ≥3.0 g/dl
Anemia for age
Elevation of alanine aminotransferase
Platetelets after 7 days ≥450,000/mm^3^
WBC ≥15,000/mm^3^
Urine WBC ≥ 10/HPF
(B)
Z score of LAD or RCA ≥2.5
Coronary arteries meet japanese Ministry of Health
Criteria for aneurysm
Internal Lumen diameter
>3mm in children <5 years old, or
>4 mm in children >5 years old
Of a segment measures ≥1.5 times that of an adjacent segment
Clearly irregular coronary lumen
Other 6 suggestive features (if ≥3 features, positive)
Perivascular brightness of coronary arteries
Lack of tapering of coronary arteries
Decreased LV function
Mitral regurgitation
Pericardial effusion
Z score in LAD or RCA of 2 to 2.5

According to the algorithm of the guidelines, assessment using laboratory tests was required and fulfilled >3 supplemental laboratory criteria (anemia for age, platelets after 7 days >45 x 10*4//vL, WBC counts >15 000/lvL and alanine aminotransferase. Therefore, we considered this patient to be suffering from incomplete Kawasaki disease was started on high dose aspirin however was not started on IV Ig because studies have shown little benefit after 10 days in preventing coronary complications and risk to benefit ratio was high. Presented case is of atypical Kawasaki disease causing dilemma to the physicians in reaching the diagnosis because of the overlapping presentations. Physicians need to recognize that overlapping viral illness can lead to the delay in diagnosis of grave conditions like incomplete Kawasaki disease, despite the use of established guidelines.

## Conflict of Interest

**None.**

Consent: JNMA **Case Report Consent Form** was signed by the patient and the original is attached with the patient chart.
